# The Utility of Phosphohistone H3 in Inter-Observer Variability of Mitotic Count in Meningioma, is There Any Benefit?

**DOI:** 10.31557/APJCP.2021.22.7.2049

**Published:** 2021-07

**Authors:** Hana Saffar, Hoda Okhovat, Saeed Arbabsoleymani, Seyed Mohammad Tavangar, Alireza Khoshnevisan, Ghazal Hajinasrollah, Zahra Hamidi Afra, Hiva Saffar

**Affiliations:** 1 *Department of Pathology, Cancer Institute, Imam Khomeini Complex Hospital, Tehran University of Medical Sciences, Tehran, Iran. *; 2 *Department of Pathology, Shariati Hospital, Tehran University of Medical Sciences, Tehran, Iran. *; 3 *Department of Community Medicine, Shahid Beheshti University of Medical Sciences, Tehran, Iran. *

**Keywords:** Phosphohistone H3-, meningioma, grade, mitosis, inter-observer variability

## Abstract

**Objective:**

One of the most reliable and decisive histologic parameters with negative prognostic impact is tumor proliferation capacity . Quantification of mitosis in H&E stained slides could be problematic and is limited by poor reproducibility and lack of objectivity. This study was designed to evaluate inter-observer variability in mitotic count using Phosphohistone H3(PHH3).

**Methods:**

Totally, 60 specimens with histologic diagnosis of meningioma were selected including 50 grade I, 7 grdae II and 3 grade III tumors. Mitotic figures were counted both in H&E stained sections and slides prepared by immunohistochemistry using Anti-Phosphohistone H3 Anti body by three observers with various level of expertise, independently.

**Results:**

Mean mitotic count by PHH3 method was higher than H&E staining for all three observers. Observer 1 and 2 revealed good correlation in mitotic count using H&E method, while observer 3 showed disagreement with both of them. However, all of them had good correlation in mitotic count using PHH3 method (cc=0.956,0.947,0.909).

**Conclusion:**

Based on our findings, PHH3 revealed good agreement between pathologists with various level of expertise and has the capability for further contribution in meningioma grading classification and specially could be beneficial for less experienced pathologists.

## Introduction

Predicting probable aggressive behavior or recurrence in meningiomas are related to various factors including location, extent of resection, invasion to brain parenchyma and histologic grade.(Elmaci et al., 2018). The three tired grading system by WHO admits risk estimation based on histomorphologic criteria (Kim et al., 2007). Among the negative known prognostic factors in meningiomas, proliferation capacity has been described as one of the most reliable and decisive histopathological parameters (Perry et al., 1997; Kim et al., 2007; Winther et al., 2016).

However, quantification of mitosis in Hematoxylin and Eosin (H&E) stained slides could be problematic (Elmaci et al., 2018; Puripat and Loharamataweethong, 2019) and is limited by poor reproducibility and lack of objectivity (Winther et al., 2016). One of the most challenges are mitosis mimickers including chromatin changes seen during apoptosis, pyknotic nuclei, necrotic or crushed cells (Hendzel et al., 1998; Ribalta et al., 2004; Winther et al., 2016; Elmaci et al., 2018; Puripat and Loharamataweethong , 2019). Moreover, heterogeneity of mitotic activity in different areas, variation in cell density and bias in sampling could be other confounding factors (Winther et al., 2016; Elmaci et al., 2018; Puripat and Loharamataweethong, 2019). 

Numerous biomarkers and methods have been evaluated for more accurate assessment of the degree of proliferation (Winther et al., 2016) .Phosphohistone H3(PHH3) is a core histone protein , together with other histones forms the major protein constituents of the chromatin in eukaryotic cells. It appears that ,PHH3 is negligible during interphase in cell cycle but reaches to maximum condensation during mitosis (Puripat and Loharamataweethong, 2019). Phosphorylation of histone H3 is mitosis specific (Fukushima et al., 2009; Olar, 2015). Immunohistochemically, it stains mitotic figures from early prophase through metaphase, anaphase and telophase (Tapia et al., 2006; Puripat and Loharamataweethong, 2019). Phosphohistone H3(PHH3) as a mitotic marker is able to distinguish mitotic cells from mitosis mimickers including apoptotic bodies, distorted and crushed cells or from necrotic and karyorrhectic debris (Puripat and Loharamataweethong, 2019). It has been proved reliable in different types of tumors (Baehner and Weidner, 2000; Colman et al., 2006; Tapia et al., 2006; Kim et al., 2007) including meningioma (Ribalta et al., 2004; Kim et al., 2007).

The alternative biomarkers, Ki67, a non-histone cell cycle protein, has been shown potential utility and been used in clinical practice (Winther et al., 2016). However, ki67, usually overestimates the proliferative activity, because cells in several phases of cell cycle, including M, G1, G2 and S are all stained positive (Puripat and Loharamataweethong, 2019). In addition, tumor infiltrating inflammatory cells also can be positive (Puripat and Loharamataweethong, 2019). So, it appears that PHH3 could be superior to ki67 in some aspects.

Considering the significance of accurate and reproducible mitotic count in grading of meningioma, this study was designed to evaluate whether mitotic count using PHH3 could decrease inter-observer variability between pathologists or not.

## Materials and Methods

**Figure 1:**
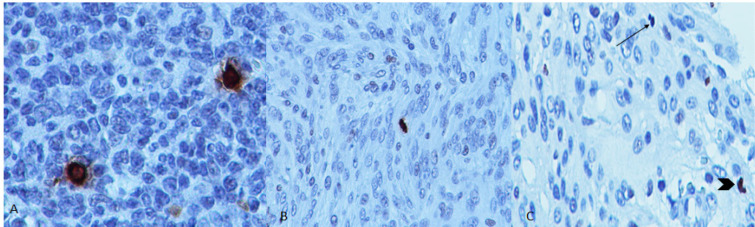
A, Two mitotic figures in tonsil tissue; B, typical mitotic figure in meningioma; C, Negative reaction of apoptotic cell with PHH3 (arrow) and positive stained non mitotic cell with intact nuclear border (arrow head)

Totally, 60 specimens with histologic diagnosis of meningioma were selected including 50 grade I, 7 grade II and 3 grade III tumors classified based on WHO classification system (Louis et al., 2016 ). The slides were reviewed and sections with the highest mitotic activity on H&E , and adequate preserved tumoral tissue were selected. Immunohistochemistry was performed using Rabbit polyclonal anti PHH3 (Biocare Medical) based on manufacturer’s protocol. Tonsil tissue was used as positive control (Figure 1).

Two pathologists (1 and 2) and one senior resident of pathology (3) reviewed the slides separately . They were blind to each other’s scoring.

The mitotic figures were counted in 10 consecutive high power fields in H&E stained slides. Then , IHC slides stained with anti PHH3 antibody were evaluated. Positive cells were counted in the same manner, at the same day or the day after.

Brown stained nuclei with loss of nuclear membrane or presence of chromosome condensation arranged along a plane or separated were considered as positive cells and intact brown stained nuclei or nuclei with smooth membrane and absence of chromosome condensation were excluded (Puripat and Loharamataweethong., 2019)(Figure 1).

All data were gathered and analyzed using SPSS version 22 software , paired sample t-test.

## Results

Sixty samples were examined. The mean age among patients in grade I, II and III were 53.3,63.14 and 69.33 years, respectively.

Mean mitotic count on H&E stained and PHH3 slides are shown in Table 1. The mean mitotic count by PHH3 method was higher for all three observers and the difference was significant for observer 2 and 3.

observer 1 and 2 revealed good correlation in mitotic count using H&E method, while observer 3 showed significant disagreement with both of them. However, all of them had good correlation in mitotic count using PHH3 method (Table 2).

Observer1 had one increase in tumor grade (upgrade : 3 mitosis/HPF to 4) and one decrease in grade( downgrade: 4 mitosis/HPF to 3) following incorporating PHH3 . Observer 2 faced two upgrades (3 mitosis/HPF to 4 and 5) when using PHH3 in comparison to H&E. However, the results for observer 3 (resident of pathology) was more variable and 4 tumors were up graded and 4 tumors down graded , totally.

**Table 1:** Comparison of Mitotic Count Using H&E and PHH3 Methods by Three Observers

Observer code	Mean mitotic count in H&E method	Mean mitotic count in PHH3 method	p value	Coefficient Correlation
1	2.40	2.65	0.066	0.854
2	2.26	2.63	0.007	0.807
3	1.76	2.66	0.000	0.762

**Table 2:** Level of Agreement and Correlation of Mitotic Count between Observers in H&E and PHH3 Methods

Observers code	H&E	PHH3
1 and 2	P=0.220;CC=0.923	P=0.811;CC=0.956
1 and 3	P=0.000;CC=0.821	P=0.811;CC=0.947
2 and 3	P=0.000;CC=0.839	P=0.709;CC=0.909

## Discussion

Meningioma constitutes about one third of the primary intracranial neoplasms. They represent the largest group of central nervous system(CNS) tumors, and have an intimate trend to recur (Puripat and Loharamataweethong, 2019). Tumor grade, proliferative capacity, histologic subtype and extent of resection are various factors influence recurrence rate (Kasuya et al., 2006; Puripat and Loharamataweethong, 2019). 

Considering mitosis as one of the most negative prognostic factors (Kim et al., 2007), accurate and precise counting of mitotic figures (MF) might be subjected to inter-observer variability. In-fact, distinguishing MF in H&E stained slides from similar chromatin changes is a subjective task (Kim et al., 2007) and this may result in inconsistencies in tumor grading specially in borderline cases (Puripat and Loharamataweethong, 2019). 

Ki67 has been widely investigated in meningioma grading. However, it is not mitosis specific and there is no agreed cut off value (Puripat and Loharamataweethong, 2019). A meta analysis from 53 studies revealed great variation in cut offs recommended to predict recurrence, ranging from 1 to 10% (Prayson, 2005; Olar et al., 2015; Elmaci et al., 2018).

PHH3 immunostaining allows the pathologist to assess morphologic features of mitosis at the same time, increasing the specificity of quantification (Ribalta et al., 2004; Bossard et al., 2006; Casper et al., 2010; Brunner et al., 2012; Elmaci et al.,2018), improvement in reproducibility and/or reduce in inter-laboratory or inter-observer variability (Angi et al., 2011; Draganova-Tacheva et al., 2013; Duregon et al., 2014; Cui et al., 2015; Duregon et al., 2015; Winther et al., 2016; Elmaci et al., 2018). Moreover, because it is mitosis specific (Aune et al., 2011; Braun et al., 2013; Olar, 2015) and stains solely mitotic cells, it appears more comparabale with mitotic index compared to ki67 which stains all phases except for G0 (Colman et al., 2006; Elmaci et al., 2018).

Fukushima et al., (2009) reviewed 45 meningioma cases classified based on WHO criteria (37 benign; 7 atypical form and 1 anaplastic). They concluded PHH3 as a reliable tool for discrimination between apoptotic and crushed cells leading to more precise counting of mitotic Figures. In addition, in their study, mitotic count based on PHH3 was significantly greater than mitotic count based on routine H&E method. By PHH3, two tumors with grade I were upgraded in to grade II (Fukushima et al., 2009; Elmaci et al., 2018). In our study, also mean mitotic count was higher in PHH3 method in comparison to H&E staining by all three observers with significant difference in two of them. 

Ribalta et al., (2004) evaluated mitotic count in 54 archival meningioma. Mean count results revealed a strong correlation between mitotic count in PHH3 and H&E methods. However, PHH3 method appeared more sensitive and there was 9 increase(up grade ) and 3 decrease (down grade) in tumor grade. Because down grading was mainly in old blocks, they explained probable loss of antigen preservation (Elmaci et al., 2018). In our study, there was two increase in grade by one of the experienced pathologists. Also one increase and one decrease in grade were reported by the other. All three up grades were in borderline cases with three mitosis in H&E. The findings probably supports more sensitivity of PHH3 method. The case with decrease in grade by PHH3 method, was a fibroblastic meningioma and probably mitotic mimickers like crushed cells were counted as mitosis in H&E method. The important point in our study was variable results in counting by our resident with four up grades and four down grades in cases after incorporating PHH3. Most of these cases were hypercellular and revealed foci with small cell morphology or increased number of apoptotic cell. We think these histologic features were the main reason for miscounting by our resident. Ribalta et al., (2004) also believes that PHH3 is particularly useful in tumors prone to error due to hypercellularity or presence of increased apoptosis (Elmaci et al., 2018). 

In a study by Duregon et al. , the validated inter and intra observer variability in 70 meningioma, using PHH3 showed good interobserver correlation in various WHO grades of tumor in comparison to usual mitotic count by H&E method. Based on their results, H&E based mitotic count had good interobserver variability but PHH3 was better (Duregon et al., 2015). We also found better agreement by PHH3 method. Moreover, we observed although H&E method could perform acceptable by more experienced pathologists, it is not as suitable for less experienced eyes ,as there was significant difference in performance of our resident in comparison with two others using H&E method, while in PHH3 method we achieved much more better agreement. So, it appears using PHH3 could add some benefits in these situations.

One of the pitfalls we encountered was that there were few positive cells in some tumors resembling leukocytes. So, we think that strict adherence to protocol (mentioned in the method section : Puripat and Loharamataweethong., 2019) is required in order not to overestimate mitotic count by PHH3 method. Baker-Grondahi et al., 2014; Elmaci et al., 2018) also reported the same finding.

Thus, mitotic count based on PHH3 staining appears a robust, easy and reliable method and could potentially decrease interobserver variability specially in less experienced pathologists. However, deciding on whether current counting method could be replaced by PHH3, requires more studies. Moreover, due to different sensitivity of various antibodies (PHH3 S10 and S28), the threshold should be standardized (Puripat and Loharamataweethong, 2019), which requires more study.

Our study has evaluated inter-observer reproducibility of mitotic count using PHH3 immunohistochemistry method among pathologists with various level of expertise. The limitation was that we did not follow the clinical outcome of the patients. Based on our findings, PHH3 has the capability for further contribution in meningioma grading classification and specially could be beneficial for less experienced pathologists.

## Author Contribution Statement

Hana Saffar: Review of slides (Pathologist), preparing the manuscript. Hoda Okhovat: Review of slides ( Resident of pathology). Saeed Arbabsoleymani: Data analysis and preparing the preliminary draft of the manuscript. Seyed Mohammad Tavangar: Final preparation and approval of manuscript. Alireza Khoshnevisan: Final preparation and approval of manuscript. Ghazal Hajinasrollah: Data analysis. Zahra Hamidi Afra: Data entry and preliminary analysis. Hiva Saffar: Study design, review the slides, final proof of manuscript and corresponding author.
